# Prevalence and multivariable predictors of breastfeeding outcomes in the United Arab Emirates: a prospective cohort study

**DOI:** 10.1186/s13006-021-00428-7

**Published:** 2021-10-12

**Authors:** Hadia Radwan, Randa Fakhry, Nick Metheny, Wegdan Baniissa, Moez Al Islam E. Faris, Reyad Shaker Obaid, Suad Al Marzooqi, Hessa Al Ghazal, Mahmoud ElHalik, Cindy-Lee Dennis

**Affiliations:** 1 Department of Clinical Nutrition and Dietetics, College of Health Sciences, Research Institute of Medical and Health Sciences, University of Sharjah, Sharjah, United Arab Emirates; 2 Department of Nursing, College of Health Sciences, Research Institute of Medical and Health Sciences, University of Sharjah, Sharjah, United Arab Emirates; 3 School of Nursing and Health Studies, University of Miami, Miami, USA; 4 Department of Psychology, United Arab Emirates University, Al Ain, United Arab Emirates; 5 Sharjah Child-Friendly Office, Sharjah, United Arab Emirates; 6 Department of Neonatology, Latifa Women and Children Hospital, Dubai, United Arab Emirates; 7 Department of Psychiatry, University of Toronto, Toronto, Canada

**Keywords:** Postpartum depression, Breastfeeding self-efficacy, Anxiety, Social support, Exclusive breastfeeding, Cohort, UAE

## Abstract

**Background:**

Despite considerable policy actions at the national and hospital levels, rates of breastfeeding in the Middle East and North Africa (MENA) region remain below the global average. There is a need to explore the modifiable factors of breastfeeding such as maternal breastfeeding self-efficacy (BSE), support, and mental health among women in this region to guide interventions in the United Arab Emirates (UAE). The aim of this study was to examine the maternal predictors of any and exclusive breastfeeding in a cohort of Emirati and expatriate women residing in the UAE with a specific focus on modifiable factors.

**Methods:**

Using a prospective cohort design, Emirati and expatriate women were recruited in the immediate postpartum period (*N* = 374) and followed at three and 6 months postpartum between February 2018 and July 2019. Questionnaires with validated tools were used to collect information on sociodemographic characteristics, breastfeeding practices, BSE, postnatal depression, and anxiety. The main outcomes in the study were Any Breastfeeding and exclusivity practices, which were assessed at three and 6 months postpartum by asking the mother about her breastfeeding behaviour during the past 7 days. Multilevel, multivariate logistic regression was used to estimate the association of different variables with breastfeeding outcomes.

**Results:**

Almost all women reported initiating breastfeeding during their stay at the hospital (*n* = 357), while only 263 (70.3%) initiated breastfeeding within the first hour of delivery. At 6 months postpartum, 301 (81.5%) women continued to breastfeed of whom 100 (26.7%) were doing so exclusively. Older mothers who initiated breastfeeding within 1 h of birth and were satisfied with the breastfeeding support they received from family and friends had significantly greater odds of any breastfeeding at 6 months. Whereas a clinically significant Edinburgh Postnatal Depression Scale (EPDS) score, low BSE score as well as employment outside the home were associated with significantly lower odds of exclusive breastfeeding and any breastfeeding at 6 months postpartum.

**Conclusion:**

This study highlights the need to develop effective education strategies and support programs targeting these modifiable variables to improve breastfeeding outcomes among women in the UAE.

## Background

Optimal breastfeeding has been described as one of the most effective interventions in reducing infant and child mortality globally [1]. The World Health Organization (WHO) and the United Nations Children’s Fund (UNICEF) recommend initiating breastfeeding within the first hour of birth with exclusive breastfeeding (EBF) for the first 6 months of life and continued breastfeeding with appropriate complementary food for up to 2 years of age or beyond [2, 3]. However, only 45% of newborns worldwide are breastfed in the first hour of life and only 38% exclusively breastfed to 6 months [4].

A woman’s ability to initiate and maintain breastfeeding is influenced by a range of modifiable and non-modifiable factors [5–7]. Extensive studies have reported associations of breastfeeding behaviors with non-modifiable factors, or factors not amenable to change via health promotion programs, such as maternal age, ethnicity, education, parity, and income [8–10]. Existing research has identified other factors such as interpersonal and intrapersonal ones which are modifiable (i.e., maternal intentions and attitudes towards breastfeeding, social support, employment policies, perceived breastfeeding self-efficacy, and emotional status) that were found to influence breastfeeding outcomes [11–14]. Focusing on modifiable factors, which are often more responsive to change than demographic and health-related variables, is fundamental when developing targeted interventions to increase breastfeeding exclusivity rates among women.

Maternal mental health, depression, anxiety, and BSE have consistently been reported as important factors influencing breastfeeding outcomes [6, 15, 16]. Mothers with poor mental health demonstrate low BSE toward both initiating and maintaining breastfeeding of their babies [5]. Maternal depression and anxiety have been found to negatively influence breastfeeding outcomes including initiation and exclusivity [17]. Previous systematic reviews have reported that women with depressive symptomatology or women with high levels of prenatal anxiety were at increased risk for negative infant-feeding outcomes, including decreased breastfeeding duration and EBF, increased breastfeeding difficulties, and decreased levels of BSE [18, 19].

Social support also has been found to be associated with breastfeeding practices as well as increasing BSE. Support to breastfeeding women can be offered by health professionals (physicians, breastfeeding consultants, social workers) or lay people (peers, family, friends) and is effective in promoting breastfeeding practices [20, 21]. A review of 34 trials with more than 29,000 mother-infant dyads in 14 countries, found that both professional and lay support increased the duration of any breastfeeding and exclusive breastfeeding [22].

Breastfeeding rates in the MENA region are below the global average. A meta-analysis of 19 studies that included countries in the MENA region revealed that breastfeeding was initiated in 34.3% of newborns within the first hour of birth but only 20.5% were exclusively breastfed for the first 6 months [23]. Similarly, in the UAE, previous studies reported suboptimal rates of initiation and EBF rates [24, 25] despite significant national policy initiatives to promote breastfeeding in the UAE including the nationwide promotion of the Baby-Friendly Hospital Initiative, the Global Strategy for Infant and Young Child Feeding, and the implementation of the International Code of Marketing of Breastmilk Substitutes [26]. It is thus important to examine the factors that influence breastfeeding practices at 6 months postpartum in the UAE.

Further national efforts and population-level strategies are required in the UAE to boost EBF rates to be in line with the UAE Vision 2021 National Agenda [27]. So, identifying the modifiable factors related to exclusive breastfeeding practices will inform targeted individual interventions that increase breastfeeding exclusivity rates in the UAE.

To our knowledge, no previous studies have examined the influence of modifiable maternal intrapersonal and interpersonal factors on infant feeding practices among women in the UAE. Therefore, the objective of this cohort study was to determine the prevalence of any breastfeeding (ABF) and EBF among women in the UAE and to identify the factors that predict breastfeeding duration and exclusivity in the first 6 months postpartum with the focus on the modifiable factors. The findings of this study will provide the needed evidence to develop and tailor interventions to complement national breastfeeding policies.

## Methods

### Study design

This was a six-month, prospective cohort study conducted in the UAE between February 2018 and July 2019. The data were collected from four out of the seven Emirates in the UAE (Abu Dhabi, Dubai, Sharjah, and Fujairah) which represented four distinct regions of the most populated Emirates in the UAE.

### Sample and setting

A convenience sample of 457 women was recruited from the maternity wards of ten hospitals in the four Emirates between February 2018 and July 2019. Detailed descriptions of the study and the recruitment methods were previously published [28]. Inclusion criteria comprised literate Emirati and expatriate women in the immediate postpartum period aged 18 to 45 years at the time of initial contact, and who had just delivered a healthy, singleton baby. Women were excluded if they had any condition that might prevent them from breastfeeding their infants, such as the presence of infant congenital disabilities.

Participants were interviewed at three time points: immediately postpartum on the maternity ward and at three and 6 months postpartum by telephone. During the first visit, a 30-min face-to-face questionnaire was completed which included sociodemographic information, questions on breastfeeding intention and previous practices, complications during pregnancy, labor and delivery, postpartum care, breastfeeding education, infant feeding method preferences, living arrangements, and family and spousal support and assistance. Follow-up questionnaires that were administered at three and 6 months postpartum included information on infant feeding, breastfeeding difficulties, parenting support, maternal and infant health data, and infant sleeping arrangements. The participants also completed a self-administered Breastfeeding Self-Efficacy Scale-Short Form (BSES-SF) [13], Edinburgh Postnatal Depression Scale (EPDS) [29], and the State-Trait Anxiety Inventory (STAI) [30].

Questionnaires at all three time points were translated from English into Arabic and were pilot tested with ten women prior to use. Women who completed the questionnaires and the tools at three and 6 months were included in this analysis (*N* = 374). There were no significant differences in demographic variables between the 374 participants included in the study and the 83 women who dropped out of the study due to loss of follow-up. Written informed consent was obtained from the participants at baseline.

#### Breastfeeding outcomes

Any Breastfeeding (ABF) and Exclusive Breastfeeding (EBF) were assessed at 6 months and measured based on the WHO criteria and followed the point-in-time method to avoid recall bias [31]. ABF included the provision of any breastmilk in the past 7 days. EBF was defined as the provision of only breastmilk, with the inclusion of vitamins and medicines, in the past 7 days. [The mothers were asked to, “Indicate if you have exclusively breastfed your infant (baby receives only breast milk, may include vitamins or medications) during all days of the past seven days”].

#### Covariates

Covariates measured at baseline or 3 months postpartum were categorized into four domains based on the empirical literature: (1) sociodemographic, (2) infant-related variables, (3) intrapersonal factors including mental health and BSE, and (4) sources of support. (1): Sociodemographic variables included maternal age (< 25, 25 – 29, > 30 years), maternal educational level (graduated from university: yes / no), maternal employment status outside the home (yes / no), Arab ethnicity (yes / no), Islamic religion (yes / no), household member smokes tobacco (yes / no), preconception maternal body mass index (BMI) > 25 (yes / no) (according to WHO classifications [32], gestational weight gain (GWG) (coded as insufficient, adequate, or excessive according to the Institute of Medicine (IOM) guidelines [33] and multiparous parity (yes / no). (2): Infant-related variables included infant sex (male or female), mode of delivery (vaginal or cesarean), skin-to-skin contact within 30 min of birth (yes / no), breastfeeding initiation within 1 h of delivery (yes / no), breastfeeding problems (e.g., cracked nipples, insufficient milk supply) (yes / no), and attended antenatal breastfeeding class (yes / no). (3): Intrapersonal variables included mental health and BSE. To measure postpartum depression, the internationally recommended 10-item EPDS was administered at all time points. A cut-off score > 9 is recommended when used as a screening measure and was used to indicate signs of clinical postpartum depression. A validated Arabic version of the tool with adequate psychometric properties was used with Arabic-speaking participants [34]. The STAI is a 40-item scale measuring two types of anxiety – state and trait – using a 4-point Likert scale. The scores range from 20 to 80 with higher scores indicating greater anxiety. A cut-off score of > 37 was used to indicate signs of clinical anxiety [35]. A validated Arabic version of the tool was used with Arabic-speaking participants [36]. Maternal confidence in breastfeeding ability was measured using the BSES-SF at baseline which is a 14-item scale with scores ranging from 14 to 70 with higher scores indicating higher levels of BSE [13].

The form was translated into Arabic to use with Arabic-speaking participants. Prior to use, a panel of experts was assembled, and the English form was translated to Arabic and back translated into English. The final approved translated version was pilot tested before it was used. (4): Sources of support covariates also included two variables indicating whether women reported that they were satisfied with the support they received from (1) their husband and (2) their friends and family at three and 6 months postpartum. This domain also included the length of maternity leave for mothers who were employed at the time of first contact (not employed, less than 3 months, 3 months, or more maternity leave).

##### Statistical analysis

Baseline descriptive statistics were compiled for all respondents and comparisons were made between the analysis sample and those excluded due to missing data using Chi-square (categorical variables) and *t*-tests (continuous variables). For the main analysis, we analysed the associations between the covariates described above and the odds of ABF and EBF at 6 months using multilevel modeling. Taking a sampling-based perspective, the nesting of respondents within hospitals and hospitals within Emirates necessitated the use of a three-level multilevel model in these analyses [34, 37, 38]. Compared to standard logistic regression analyses, multilevel modeling corrects for the downward bias in standard errors created by the non-independent nature of nested data and introduces an error term that captures the effects of unobserved covariates [37, 39].

Two identical three-level logistic regression models were fitted using all covariates described above, one for each of the two outcome variables, with hospital and Emirate as random intercepts. Sensitivity analyses were fitted using two-level and single-level models to ensure severe bias did not result from the small number of level-two and level-three units. Data cleaning, management, and analyses were conducted in Stata, version 16 (STATA Corporation, 2018, College Station, TX).

## Results

### Sample characteristics

**Fig. 1:**
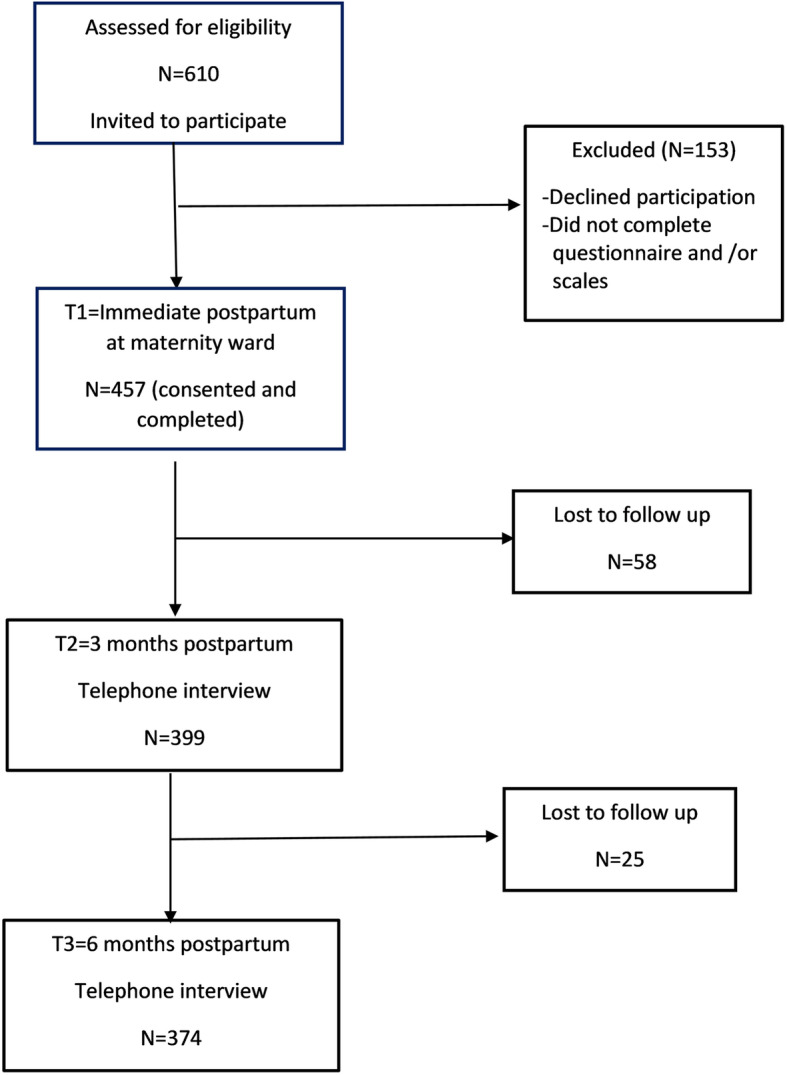
Flow chart of the study timeline and participation

Out of 610 mothers who were invited to participate, 457 accepted to enroll and completed the visit 1 questionnaire (74.9%). For the follow-up visit at 3 months postpartum, only 399 responded (87.3%). At 6 months postpartum, 374 mothers completed visit 3 questionnaires and were included in the analysis for this study (response rate 81.8%) (Fig. 1).

Table 1 shows the descriptive statistics of the total number of participants included in this study (*N* = 374). The majority were Muslim (80.5%, *n* = 301) and of Arabic origin (70.9%, *n* = 265). Women were on average 31.2; (SD = 5.5) years old, and most were multiparous (77%, *n* = 288). While nearly three-quarters of the women had graduated from university (72%, *n* = 268), only 39% (*n* = 146) were employed outside the home, and 67.8% (*n* = 99) of the working mothers had a maternity leave of ≥3 months. Less than half of the women were delivered by cesarean section (41.4%, *n* = 155), while 84.5% (*n* = 316) had skin-to-skin contact with their infants. As for the pre-pregnancy BMI, 49.5% (*n* = 185) were either overweight or obese before pregnancy, and 38.5% (*n* = 144) gained excessive GWG during pregnancy.

**Table 1:** Demographic characteristics, mental health, and social support of the study participants (*N* = 374)

Variable	n (%)/Mean ± SD*
*Demographic variables*	
Mother age (year)	31.2 ± 5.5
18 – 24.9	44 (11.7)
25 – 29.9	96 (25.7)
≥ 30	234 (62.6)
Maternal education	
Less than a university degree	106 (28.3)
Has a university degree	268 (71.7)
Mother employment	
Not working	228 (61.0)
Working (full or part-time)	146 (39.0)
Maternal leave	
< 3 months	47 (32.2)
≥ 3 months	99 (67.8)
Mother nationality	
Emirati	144 (38.5)
Other Arab	121 (32.4)
Asian	89 (23.8)
Westerners/Other	20 (5.3)
Religion	
Muslim	301 (80.5)
Christian	54 (14.4)
Hindu	19 (5.1)
Pre-pregnancy BMI (kg/m^2^) ^*^	
< 25	189 (50.5)
≥ 25	185 (49.5)
Gestational weight gain	
Adequate	128 (34.2)
Insufficient	102 (27.3)
Excessive	144 (38.5)
Parity	
Primiparous	86 (23.0)
Multiparous	288 (77.0)
Smoking among family members	
No one in the household smokes tobacco	248 (66.3)
At least one person in the household smokes tobacco	126 (33.7)
*Birth and intrapartum variables*	
Infant sex	
Male	191 (51.1)
Female	183 (48.9)
Type of delivery	
Vaginal	219 (58.6)
Cesarean	155 (41.4)
Post-delivery skin-to-skin contact	
Yes	316 (84.5)
No	58 (15.5)
Initiation of breastfeeding	
< 1 h	263 (70.3)
≥ 1 h	94 (25.1)
Breastfeeding problems reported	
Yes	156 (41.7)
No	218 (58.3)
Attended breastfeeding class education	
Yes	284 (75.9)
No	90 (24.1)
*Mental health and self-efficacy*	
Clinically significant EPDS^a^ score	
Yes	86 (23.0)
No	288 (77.0)
Clinically significant STAI^b^ score	
Yes	253 (67.6)
No	121 (32.4)
Breastfeeding self-efficacy scale-short form	52.7 ± 11.4
*Sources of breastfeeding support*	
Satisfaction with support from husband	
Yes	351 (93.9)
No	23 (6.1)
Satisfaction with support from family and friends	
Yes	341 (91.2)
No	33 (8.8)
*Breastfeeding behavior*	
ABF^c^ at 3 months postpartum (*n* = 399)	
Yes	372 (93.2)
No	27 (6.8)
EBF^d^ at 3 months postpartum (n = 399)	
Yes	201 (50.4)
No	198 (49.6)
ABF at 6 months postpartum (n = 374)	
Yes	301 (80.5)
No	73 (19.5)
EBF at 6 months postpartum (n = 374)	
Yes	100 (26.7)
No	274 (73.3)

*Body Mass Index (kg/m^2^)

^a^Edinburgh Postnatal Depression Scale

^b^State-Trait Anxiety Inventory

^c^Any breastfeeding

^d^Exclusive breastfeeding

Results related to maternal mental health showed that 23% (*n* = 86) of women reported clinically significant postpartum depression scores on the EPDS scale, and 67.6% (*n* = 253) reported clinically significant anxiety scores on the STAI scale. The majority of women were content with the breastfeeding support they received from family and friends (91%, *n* = 341), and 94% (*n* = 351) reported satisfaction with the breastfeeding support received from their husbands. The mean BSES-SF score reported by the mothers at immediate postpartum was 52.7; (SD = 11.4).

### Prevalence of any breastfeeding and exclusivity

Almost all of the women reported initiating breastfeeding during their stay at the hospital (*n* = 357), while only 70.3% (*n* = 263) put their infants to their breast within the first hour of delivery. At 3 months, 93.2% (*n* = 372/399) of women continued to breastfeed of which 50.4% (*n* = 201/399) were doing so exclusively. At 6 months, 80.5% (*n* = 301/374) continued to breastfeed and 26.7% (*n* = 100/374) did so exclusively (Table 1).

#### Predictors of breastfeeding outcomes at six months

Results of the multilevel logistic regression for breastfeeding behaviors at 6 months are reported in Table 2. Among sociodemographic factors, women aged 25 – 29.9 were significantly more likely to continue to breastfeed than younger or older women [Adjusted Odds Ratio (AOR) 3.09; 95% CI 1.12, 8.48; *p* < 0.028]. Employed women were less likely to continue to breastfeed in comparison to those who were not working (AOR 0.43; 95% CI 0.22, 0.8; *p* < 0.013). Moreover, women who initiated breastfeeding within 1 hour of delivery were three times more likely to continue to breastfeed to 6 months (AOR 3.20; 95% CI 1.55, 6.62; p < 0.01). Women who had a high BSE in the immediate postpartum period were over 4 times more likely to continue to breastfeed to 6 months in comparison to those who lacked confidence in their breastfeeding ability (AOR 4.50; 95% CI 1.58, 12.85; *p* < 0.003). Moreover, those who reported clinically significant EPDS scores at 6 months postpartum were less likely to continue to breastfeed (AOR 0.51; 95% CI 0.26, 0.99; *p* < 0.038). On the other hand, maternal anxiety did not influence breastfeeding duration. Women who reported a high level of satisfaction with breastfeeding support from family and friends were significantly more likely to continue to breastfeed to 6 months (AOR 3.70; 95% CI 1.34, 10.21; *p* < 0.008) whereas support from their husbands had no influence.

**Table 2:** Results of multilevel logistic regression models for variables associated with breastfeeding behaviors at six months (*n* = 374)

	ABF^a^ at 6 months			EBF^b^ at 6 months		
	AOR	(95% CI)	***P***-value	AOR	(95% CI)	***P***-value
**Age (year)**						
< 25 years		1			1	
25 – 29.9 years	**3.09**	**(1.12, 8.48)**	**0.028**	1.63	(0.58, 4.84)	0.373
≥ 30 years	1.90	(0.75, 4.80)	0.178	1.59	(0.56, 4.51)	0.383
**Mother has a university degree**						
No		1			1	
Yes	0.96	(0.46, 2.03)	0.819	1.28	(0.69, 2.69)	0.515
**Mother employed**						
No		1			1	
Yes	**0.43**	**(0.22, 0.83)**	**0.013**	**0.28**	**(0.14, 0.54)**	**0.001**
**Mother nationality**						
Arab		1			1	
Others (westerner, Asian, African)	2.67	(070, 10.11)	0.146	1.73	(0.62, 4.89)	0.29
**Religion**						
Muslim		1			1	
Other	2.44	(0.61, 10.81)	0.220	1.43	(0.54, 4.08)	0.483
**Anyone in the household smokes tobacco**						
No		1			1	
Yes	0.57	(0.30, 1.07)	0.082	0.63	(0.34, 1.15)	0.134
**Pre-pregnancy BMI** (kg/m^2^)^**c**^						
< 25		1			1	
≥ 25	0.61	(0.32, 1.16)	0.132	1.20	(0.65, 2.22)	0.528
**Gestational weight gain**						
Insufficient (below recommendations)	1.40	(0.60, 3.16)	0.408	1.00	(0.48, 2.07)	0.999
Adequate		1			1	
Excessive (above recommendations)	2.12	(0.99, 4.44)	0.545	1.18	(0.60, 2.23)	0.614
**Parity**						
Primiparous		1			1	
Multiparous	1.49	(0.64, 3.60)	0.375	1.42	(0.65, 3.02)	0.388
**Infant sex**						
Male		1			1	
Female	0.64	(0.33, 1.12)	0.115	1.01	(0.59, 1.75)	0.959
**Type of delivery**						
Vaginal		1			1	
Cesarean	1.16	(0.58, 2.33)	0.676	1.08	(0.57, 2.05)	0.773
**Skin-to-skin contact within 30 min of birth**						
No		1			1	
Yes	0.46	(0.19, 1.11)	0.112	1.71	(0.67, 4.20)	0.247
**Breastfeeding initiated within one hour of birth**						
No		1			1	
Yes	**3.20**	**(1.55, 6.62)**	**0.01**	1.06	(0.51, 2.21)	0.863
**Breastfeeding problems**						
No		1			1	
Yes	0.48	(0.23, 1.01)	0.530	0.62	(0.34, 1.18)	0.133
**Attended breastfeeding class**						
No		1			1	
Yes	1.16	(0.56, 2.40)	0.688	0.88	(0.46, 1. 75)	0.725
**Breastfeeding self-efficacy**						
Bottom 75%		1			1	
Top 25%	**4.50**	**(1.58, 12.85)**	**0.003**	**2.31**	**(1.20, 4.44)**	**0.012**
**Clinically significant EPDS**^**d**^ **score at any time point**						
No		1			1	
Yes	**0.51**	**(0.26, 0.99)**	**0.038**	**0.39**	**(0.22, 0.71)**	**0.002**
**Clinically significant STAI**^**e**^ **score at any time point**						
No		1			1	
Yes	0.54	(0.18, 1.61)	0.259	0.83	(0.39, 2.13)	0.834
**Satisfied with breastfeeding support from husband**						
No		1			1	
Yes	1.28	(0.38, 4.30)	0.680	1.32	(0.35, 5.06)	0.698
**Satisfied with breastfeeding support from family and friends**						
No		1			1	
Yes	**3.70**	**(1.34, 10.21)**	**0.008**		1.27 (0.42, 3.82)	0.692

^a^AOR Odds Ratio values adjusted by all the variables included in this model after performing the multivariate analysis

^a^Any breastfeeding, ^b^ Exclusive breastfeeding, ^c^ Body mass index, ^d^ Edinburgh Postnatal Depression Scale, ^e^ State-Trait Anxiety Inventory

Regarding breastfeeding exclusivity, maternal employment, BSE, and EPDS were associated with EBF. Employed mothers were less likely to exclusively breastfeed at 6 months (AOR 0.28;(95% CI 0.14, 0.54; *p* < 0.001). Women with a high level of BSE were twice as likely to report exclusive breastfeeding at 6 months (AOR 2.31; 95% CI 1.20, 4.44; *p* < 0.012). While those with depressive symptomatology were significantly more likely to have discontinued exclusive breastfeeding (AOR 0.39; 95% CI 0.22, 0.71; *p* < 0.002).

## Discussion

This cohort study is the first multi-Emirate investigation to examine prospectively breastfeeding outcomes and factors that influence duration and exclusivity in the UAE. In this study, 95% of women reported initiating breastfeeding and 70% did so within the first hour following delivery. Even though 80% of women were still breastfeeding at 6 months, only 26.7% of all mothers were doing so exclusively. The significant predictors of continued breastfeeding up to 6 months included maternal age, unemployment status, initiation of breastfeeding within the first hour of delivery, high BSE, no postpartum depressive symptoms, and high-perceived breastfeeding support from family and friends. As for EBF at 6 months, only three factors were predictive: unemployment status, high BSE in the immediate postpartum period, and no postpartum depressive symptoms at 3 months postpartum.

Approximately 27% of the infants in this study were exclusively breastfed to 6 months. Previous research in the UAE reported similar low breastfeeding exclusivity rates [24]. A similar rate was reported by a recent study in Saudi Arabia (28%) [40]. This rate is slightly higher than what was reported by a systematic review of breastfeeding rates in the Middle East region of 20.5% (CI: 14.5% – 28.2%) of pooled prevalence rate for exclusive breastfeeding at 6 months [23]. However, a recent study in Lebanon has reported a higher EBF rate (32%) [41] as well as in Turkey (38.9%) [42]. These findings indicate that women across the Middle East, including the UAE, are still far from meeting the 2025 WHO recommendation of 50% exclusive breastfeeding at 6 months. Our findings are disappointing given that the UAE has invested considerable efforts into promoting exclusive breastfeeding since 2014 [26].

The UAE government has undertaken several WHO initiatives including incentivizing all government hospitals and the majority of private hospitals to become certified according to the Baby-Friendly Hospital Initiative and recently adopted The International Code of Marketing of Breast-milk Substitutes in 2018. The largely successful uptake of these national initiatives may explain why 70% of the women in this study initiated breastfeeding within 1 h of delivery and provided skin-to-skin contact. This rate was higher than the global average initiation rate (45%) [4] and even higher than what was reported earlier in a study in Abu Dhabi (59.8%) and Saudi Arabia [43]. Regionally, a meta-analysis found that only 34.3% of newborn infants in the MENA region received breastmilk within 1 h of birth [23]. However, the early initiation rate in our study was significantly associated with increased breastfeeding duration but not with EBF.

Similar findings were reported in other studies [44, 45] where it was explained that the reason for less EBF despite high initiation rate among their participants was due to many factors such as employment and deficient knowledge and education about exclusive breastfeeding benefits to mother and infant. So, despite this initial supportive breastfeeding environment in UAE hospitals, our results clearly indicated that hospital policies based on national recommendations are not sufficient to maintain EBF to 6 months postpartum.

More efforts are needed to support mothers to exclusively breastfeed beyond the hospital and the clinical setting. and to address other modifiable intrapersonal and interpersonal factors if national breastfeeding targets are to be improved.

In contrast to previous research studies primarily conducted in Western countries [46], in this study, few sociodemographic factors were predictive of breastfeeding duration and exclusivity at 6 months. The only significant demographic variable among UAE women predictive of exclusivity was maternal unemployment. This finding is consistent with previous research, which suggested that returning to the workplace is one of the most common reasons globally as to why women discontinue breastfeeding [47]. In 2019, the UAE Human Resources Law in Federal Government was passed where, “women are entitled to three months of fully paid maternity leave”. After the female employee resumes work, she is entitled to 2 hours per day as breastfeeding leave for 6 months and then to 1 h for the next 6 months. This still fell short of the International Labour Organization’s recommendation of 18 weeks of maternity leave. The extension of maternity leave to 3 months is encouraging and assists women to breastfeed exclusively and for a prolonged duration [48].

In our study, the majority of women had a three-month maternity leave or more (68%) which might explain the drop in the EBF rate from 54% at 3 months to 27% at 6 months. Our findings highlighted the need for national policies that support the provision of longer maternity leave and mother-friendly workplaces that enable women to be close to their infants to support continued breastfeeding and provide adequate nursing facilities such as space for mothers to pump their milk and properly store it. In a recent systematic review of 22 studies from ten different countries that include both public- and private-sector organizations, providing a breastfeeding space was the most common employer-based support studied, followed by breastfeeding breaks and comprehensive breastfeeding support programs [49]. These supportive strategies could be more widely adopted in the UAE.

In this study, women who had high BSE in the immediate postpartum period were significantly more likely to be exclusively breastfeeding at 6 months. Consistent with previous research, BSE is predictive of breastfeeding outcomes. Importantly, two systematic reviews have clearly demonstrated that interventions targeting BSE significantly improve breastfeeding outcomes [50, 51]. Health professionals are urged to screen and improve mothers’ breastfeeding confidence which in turn will enable them to overcome inevitable difficulties and promote continued breastfeeding. Our findings add to the growing evidence regarding the effectiveness of BSE interventions as a modifiable variable that practitioners can target to improve breastfeeding rates among women in the UAE.

Our study also found that women with depressive symptoms within the first 3 months postpartum were significantly less likely to continue to breastfeed to 6 months and do so exclusively. This is consistent with previous reviews suggesting that maternal depression significantly influences breastfeeding outcomes including increased difficulties [18, 19]. Current practices pertaining to the management of perinatal mental health in the UAE are limited. Screening alone for maternal depression and anxiety is insufficient and should be accompanied by an evidence-based intervention program for early detection and management. One particular intervention that has recently received considerable attention is the “Program in Support of Moms (PRISM)” which aims to close gaps in health care delivery systems to ensure that women with depression, during and after pregnancy, receive quality treatment [52]. Tailoring PRISM to the Emirati context may be one way to increase EBF via improved maternal mental health.

Breastfeeding decisions are continually being made within a woman’s social and community context that includes influences from extended family, friends, media images, direct marketing and advertising, local and national policies, and prevailing cultural and religious norms. In the current study, perceived support from family and friends was a significant predictor of breastfeeding at 6 months, a finding consistent with previous research [12, 53]. Support from other women has been found to not only increase BSE via verbal persuasion and vicarious experiences but peer (mother-to-mother) support has also been found to prevent postpartum depression [54]. Interestingly, support from the husband did not influence breastfeeding outcomes in this study, which is inconsistent with studies conducted in Western countries [55, 56]. However, in many MENA contexts, influence from mothers, mothers-in-law, and other female family members and friends tends to supplant spousal influence regarding traditionally gendered activities, such as breastfeeding [53, 57]. Therefore, interventions targeting female support networks of new mothers may be particularly helpful in improving EBF.

This study has numerous strengths including the longitudinal design which examined prospectively the infant feeding practices and the psychosocial factors until 6 months postpartum. The close regular follow-up with the mothers minimized recall bias. Moreover, the instruments used in this study were culturally appropriate and validated and allowed the early identification of women at risk of breastfeeding discontinuation.

There are a few limitations that should be noted. The main limitations of the current study include the selection bias and loss to follow-up, typical of longitudinal studies. This was not a probability sample and thus cannot rule out selection bias. Another limitation could be that the participants were recruited during the postpartum period so the opportunity to measure maternal mental health was limited to postpartum, and we did not ask about past mental history which we could have adjusted for.Moreover, since the majority of our population were expatriates, our results should be generalized only with great care. There is also strong social desirability inherent in questions about breastfeeding, potentially introducing bias. We also chose to dichotomize several variables due to small cell sizes, potentially reducing power and obfuscating meaningful differences between categories. A larger sample size would have allowed for more nuanced analysis and increased the generalizability of the study results.

## Conclusions

This study represents the first cohort study to investigate multilevel drivers of breastfeeding behaviors in the UAE. Several important modifiable factors for sub-optimal breastfeeding were identified in this study: BSE, maternal postpartum depression, employment, and social support from family and friends. Results indicate that UAE health authorities’ efforts to promote exclusive breastfeeding should move beyond hospital settings and should include multifaceted interventions to maintain EBF throughout the first 6 months postpartum. Early identification of likely depressive symptoms using the EPDS, as well as perinatal assessment of BSE using the BSES-SF and subsequent referral for formal antenatal or postnatal risk assessment could guide intervention programs to improve EBF rates in the UAE. Moreover, community-based groups need to include family members such as husbands, grandmothers, sisters, friends, etc. in education and support programs for breastfeeding. Understanding those modifiable factors that influence breastfeeding exclusivity for 6 months will provide public health specialists the opportunity to tailor policy and practice in protecting, promoting, and supporting breastfeeding in high-risk mothers. This will lead to improved maternal, child, and societal health outcomes for this young and rapidly changing society.

## Data Availability

The datasets used and/or analyzed during the current study are available from the corresponding author on reasonable request.

## Abbreviations

ABF: Any Breastfeeding
BSE: Breastfeeding self-efficacy
BMI: Body mass index
BSES-SF: Breastfeeding Self-Efficacy Scale – Short Form
EBF: Exclusive breastfeeding
EPDS: Edinburgh Postnatal Depression Scale
GWG: Gestational weight gain
IOM: Institute of Medicine
MENA: The Middle East and North Africa
STAI: State-Trait Anxiety Inventory
UAE: United Arab Emirates
WHO: World Health Organization
